# Myelodysplastic Syndrome Secondary to Chimeric Antigen Receptor T-cell (CAR-T) Therapy for the Treatment of Relapsed/Refractory Multiple Myeloma

**DOI:** 10.7759/cureus.103706

**Published:** 2026-02-16

**Authors:** Waqqas Tai, Daniel Kim, Joe Dib, Jessica Thomas, Swathi Gopishetty, Precious Idogun, Afoma Anyadibe, Anuoluwa Ayetoran, Syed Zaidi, Savitha Balaraman, Ishmael Jaiyesimi

**Affiliations:** 1 Hematology and Oncology, Corewell Health William Beaumont University Hospital, Royal Oak, USA; 2 Hematology and Oncology, Kansas City University, Kansas City, USA; 3 Pathology, Corewell Health William Beaumont University Hospital, Royal Oak, USA

**Keywords:** car-t cells in hematological malignancies, idecabtagene vicleucel, ide-cel, myelodysplastic syndrome (mds), plasma cell dyscrasias, relapsed/refractory multiple myeloma, therapy-related mds

## Abstract

We present the case of a 65-year-old man with relapsed/refractory multiple myeloma (RRMM) who developed therapy-related myelodysplastic syndrome (MDS) following chimeric antigen receptor T-cell (CAR-T) therapy with idecabtagene vicleucel (ide-cel). The patient, initially diagnosed in 2019, underwent multiple lines of therapy, including high-dose melphalan with autologous stem cell transplantation, before receiving ide-cel for progressive disease. Despite initial remission, a day 100 bone marrow biopsy revealed MDS with a 7q deletion, a cytogenetic abnormality not present on prior marrow evaluations. While CAR-T therapy has revolutionized RRMM treatment, long-term complications such as therapy-related hematologic malignancy remain under-recognized.

The temporal relationship between CAR-T infusion and the emergence of a new therapy-induced cytogenetic abnormality raises concern for either CAR-T stress revealing a pre-existing myeloid clone or acceleration of therapy-induced myeloid neoplasia in a heavily pretreated patient. This case illustrates the need for standardized guidelines for malignancy screenings and long-term monitoring in CAR-T recipients. Further studies are essential to delineate the timing, mechanisms, and risk factors for secondary malignancies in CAR-T recipients.

## Introduction

Relapsed/refractory multiple myeloma (RRMM) refers to a disease that progresses after achieving at least a minimal response to prior therapy or progresses within 60 days of the most recent treatment, including disease unresponsive to salvage therapy [1]. Standard treatment strategies include autologous stem cell transplantation and combination regimens incorporating immunomodulatory drugs (IMiDs), proteasome inhibitors (PIs), anti-CD38 monoclonal antibodies, and bispecific T-cell engagers (BiTEs) [2]. Although these therapies have substantially improved outcomes, multiple myeloma remains incurable, and the management of RRMM continues to represent a major clinical challenge, highlighting the need for more durable and effective treatment strategies [2].

Over the past decade, novel therapies for RRMM have evolved substantially with the introduction of B-cell maturation antigen (BCMA) directed therapies, most notably chimeric antigen receptor T-cell (CAR-T) therapy. Two FDA-approved BCMA-targeted CAR-T products, ciltacabtagene autoleucel (cilta-cel) and idecabtagene vicleucel (ide-cel), are approved for treatment in later lines [3]. BCMA is uniformly expressed on myeloma cells with minimal expression outside plasma and mature B cells, making it an ideal target with limited off-tumor toxicity [4]. Ide-cel binds a single BCMA domain, while cilta-cel targets two [5]. While these therapies have transformed outcomes in RRMM, their long-term hematologic consequences remain incompletely characterized, including the potential development of secondary myeloid neoplasms such as myelodysplastic syndrome (MDS). 

## Case presentation

**Figure 1:**
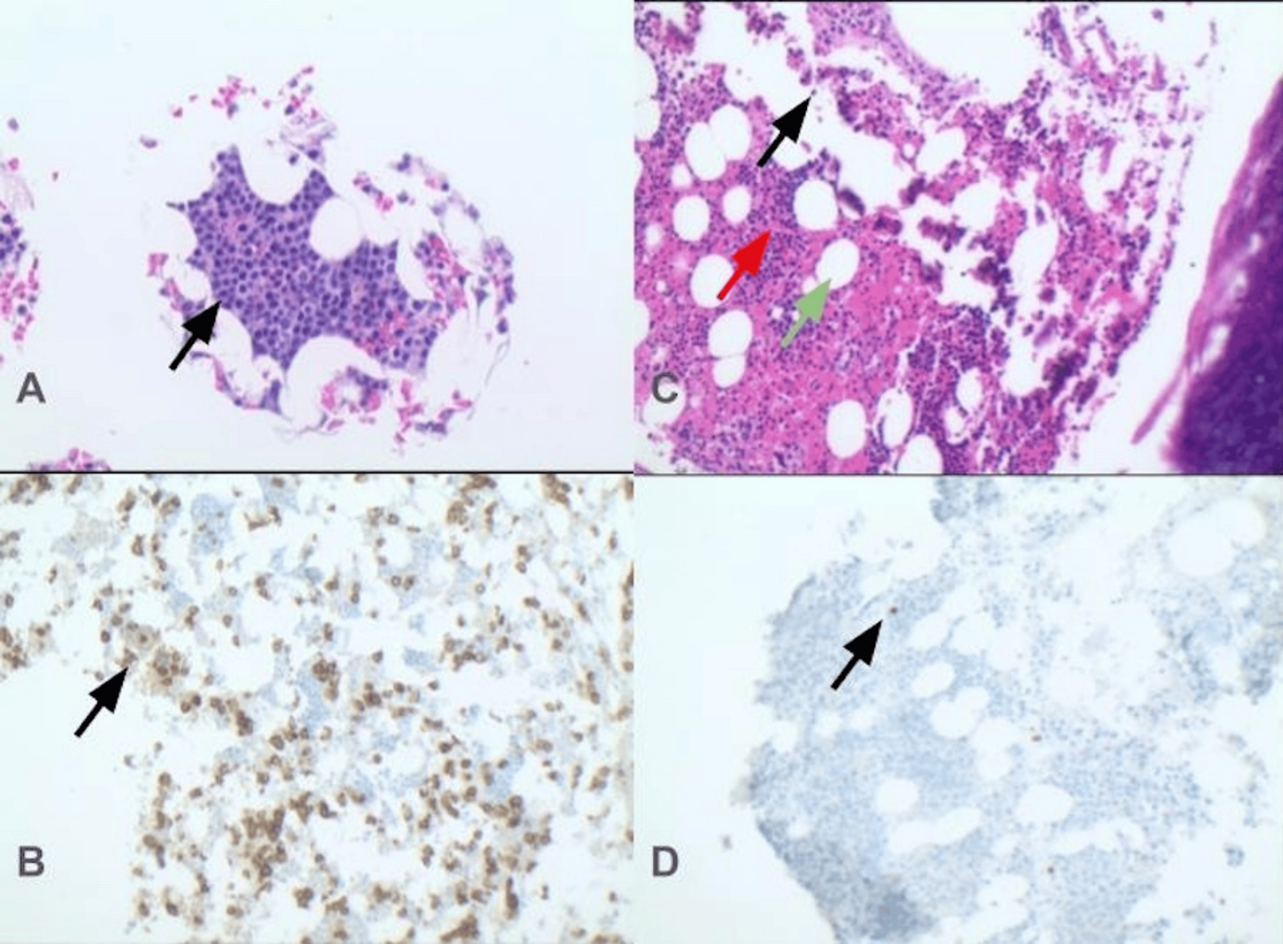
Pre-CAR-T vs post-CAR-T bone marrow biopsy and immunohistochemistry. (A) Core biopsy demonstrating extensive plasma cell infiltration. Arrow pointing to a plasma cell (B) CD138 immunostaining highlighting neoplastic plasma cells consistent with multiple myeloma. (C) Post-CAR-T therapy marrow showing trilineage hematopoiesis (black arrow: maturing myeloid precursors; red arrow: erythroid islands; green arrow: adipocytes). (D) CD138 staining post-CAR-T demonstrating only sparse residual plasma cells, supporting remission. CAR-T: chimeric antigen receptor T-cell

This is the case of a 65-year-old man with a significant past medical history of chronic lower back pain status post kyphoplasty and hypercalcemia of unknown etiology. In the summer of 2019, he presented to the hospital with severe, intractable lower back pain and worsening mentation. Initial labs revealed normocytic anemia with a hemoglobin of 9.3 g/dL, acute kidney injury (AKI) with a creatinine of 3.85 mg/dL, and a calcium level of 10.5 mg/dL. Given concern for multiple myeloma, myeloma-defining studies were obtained. An XR bone survey identified a 4 cm lytic lesion in the right iliac bone. Serum protein electrophoresis (SPEP) showed the following: free kappa 97.6 mg/L, free lambda 0.49 mg/L, kappa/lambda ratio 199.18, and beta-2 microglobulin 15.53 mg/L, lactate dehydrogenase (LDH) 103 U/L, and albumin 3.7 g/dL. Karyotype and myeloma fluorescence in situ hybridization (FISH) panel were normal (Table 1). An image-guided bone biopsy confirmed a plasma cell neoplasm, which was further supported by a bone marrow biopsy showing plasma cell myeloma with up to 70% plasmacytosis (Figure 1). The complete workup confirmed that the patient had Multiple Myeloma Revised International Staging System (R-ISS) stage II. 

**Table 1:** Labs obtained at the time of initial diagnosis in 2019. This table includes all major myeloma defining labs. Hb: hemoglobin; MCV: mean corpuscular volume; AKI: acute kidney injury; LDH: lactate dehydrogenase; FISH: fluorescence in situ hybridization

Lab	Result	Reference range	Units
Hb	9.3	13.5-17.5 (male)/12.0-15.5 (female)	g/dL
MCV (normocytic)	Within normal range	80-100	fL
Creatinine	3.85	0.6-1.2	mg/dL
Calcium	10.5	8.5-10.2	mg/dL
Albumin	3.7	3.5-5.0	g/dL
LDH	103	140-280	U/L
Free kappa light chains	97.6	3.3-19.4	mg/L
Free lambda light chains	0.49	5.7-26.3	mg/L
Kappa/lambda ratio	199.18	0.26-1.65	Ratio
Beta-2 microglobulin	15.53	0.7-1.8	mg/L
Bone marrow plasma cell percentage	Up to 70%	<5%	%
Karyotype	Normal	-	-
Myeloma FISH panel	Normal	-	-
Bone lesion (iliac, lytic)	4	-	cm

The patient was initially treated with lenalidomide, bortezomib, and dexamethasone. He demonstrated normalization of the free light chains of 0.91/0.65 mg/dL with a ratio of 1.40, supporting an appropriate response to first-line therapy. However, insufficient data is available at this time to determine the International Myeloma Working Group (IMWG) response due to limited access to records. The patient subsequently underwent autologous stem cell transplantation. Post-transplant, he was started on lenalidomide maintenance for 15 cycles but was unable to tolerate therapy due to thrombocytopenia and was transitioned to biweekly bortezomib maintenance until disease progression. In 2022, a bone marrow biopsy was performed for worsening pancytopenia. The biopsy confirmed relapsed disease, and treatment was switched to carfilzomib, daratumumab, and dexamethasone, followed by daratumumab maintenance. In 2023, a repeat bone marrow biopsy confirmed further relapse, and he was switched to cyclophosphamide, pomalidomide, and dexamethasone. Several months later, due to continued disease progression, he was started on carfilzomib, selinexor, and dexamethasone, but was unable to tolerate it due to significant thrombocytopenia.

**Figure 2:**
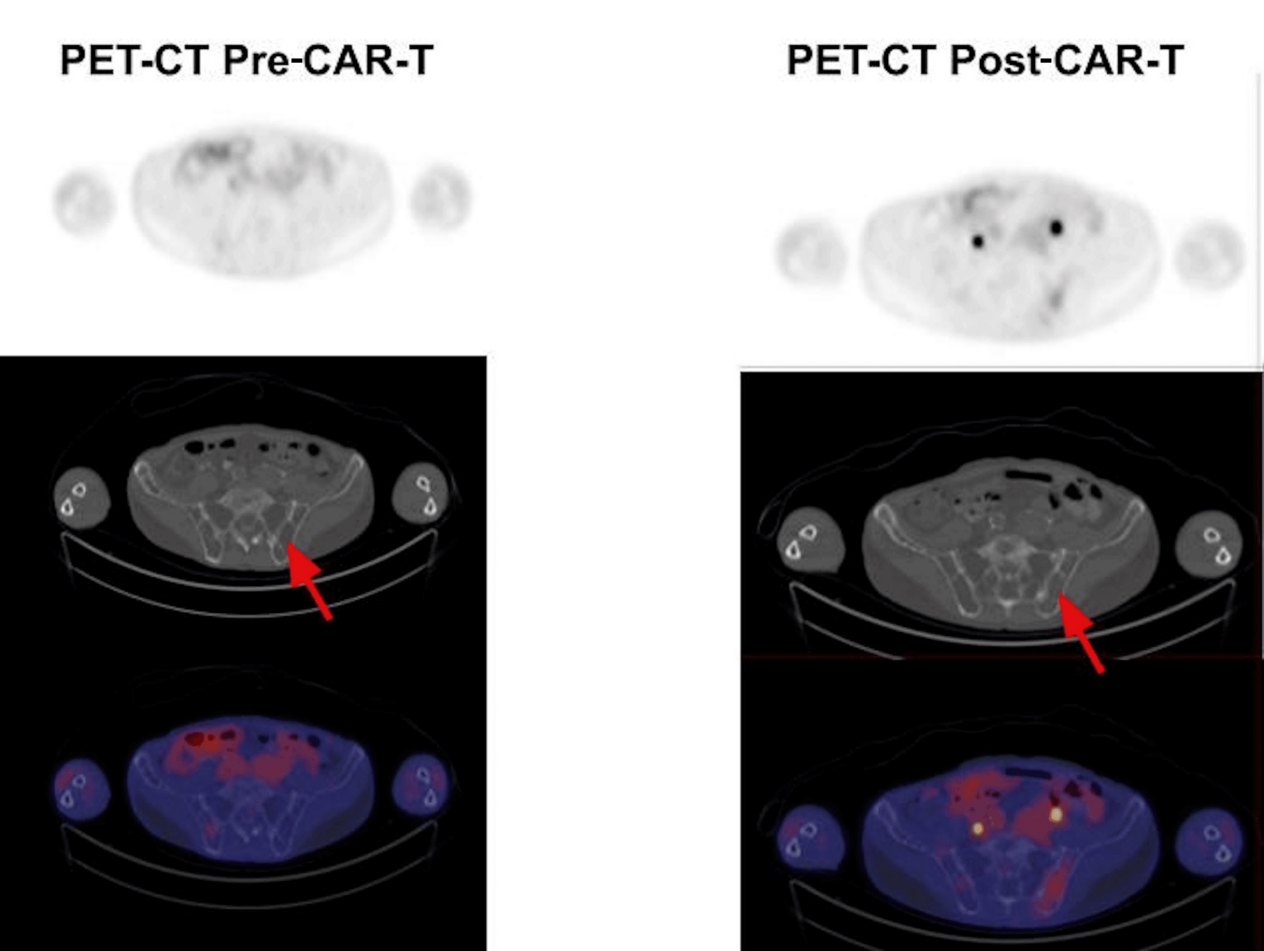
Pre- and post-CAR-T PET-CT scan. No abnormal focal activity observed, all below hepatic SUV. Diffuse lytic and mottled appearance of the osseous structures (red arrow). No focal activity within the osseous structures suggesting metabolic active disease. PET-CT: positron emission tomography-computed tomography; CAR-T: chimeric antigen receptor T-cell; SUV: standardized uptake value

**Figure 3:**
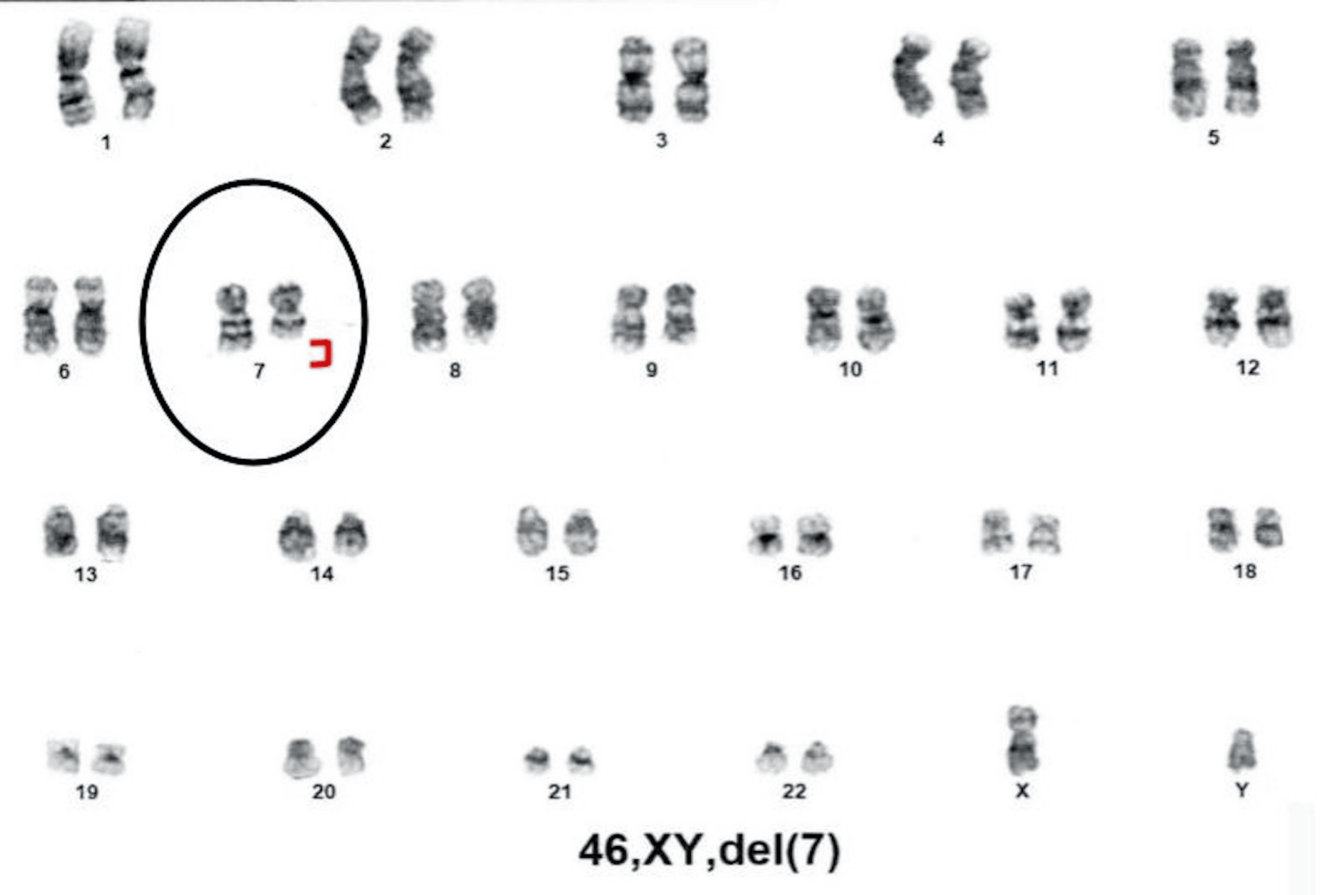
Conventional karyotype demonstrating the deletion of the long arm of chromosome 7 (46,XY,del(7)), with chromosome 7 highlighted by black circle and deletion indicated by red bracket.

In May 2024, a bone marrow biopsy showed plasma cells comprising approximately 10% of the marrow cellularity. No significant dyspoiesis was identified in any lineage. Concurrent flow cytometry showed kappa monotypic plasma cells consistent with RRMM. Next-generation sequencing (NGS) was unremarkable. Given the failure of four prior lines of therapy, the patient was offered ide-cel for RRMM. In July of 2024, the patient completed a three-day course of lymphodepleting chemotherapy with cyclophosphamide 300 mg/m² IV and fludarabine 30 mg/m² IV and then subsequently underwent ide-cel infusion. His hospital course was complicated by grade 1 cytokine release syndrome (CRS), which resolved, and he was later discharged home in stable condition, doing well with his activities of daily living (ADLs). The routine day 100 positron emission tomography-computed tomography (PET-CT) scan showed no suspicious focal fluorodeoxyglucose (FDG) activity in the neck, chest, abdomen, or pelvis (Figure 2). However, a subsequent routine post-CAR-T bone marrow biopsy revealed moderately hypocellular bone marrow with trilineage hyperplasia associated with pancytopenia (Figure 1). Karyotyping showed a 7q deletion, indicating the presence of a therapy-related myelodysplastic clone within the bone marrow (Figure 3). This is consistent with an emergence of a new dysplastic clone. Review of prior bone marrow examinations, including the biopsy immediately before CAR-T, did not demonstrate morphologic dysplasia, blasts, or other cytogenetic abnormalities suggestive of MDS. NGS was sent; only findings of significance were TP53 variant of unknown significance (VUS). The patient has been referred back to his primary oncologist to discuss a tentative chemotherapy plan for his MDS, due to an International Prognostic Scoring System (IPSS) score of 4.

## Discussion

The KarMMa trials established the efficacy and safety of ide-cel [6-8]. In the phase 2 trial, comprising 140 heavily pretreated patients, the overall response rate was 73%, including 33% complete responses and 26% minimal residual disease (MRD)-negative remissions [7]. The phase 3 international study of 386 patients showed superior progression-free survival with ide-cel compared to standard therapy (13.3 vs. 4.4 months) [8]. Neutropenia and immune effector cell-associated neurotoxicity syndrome (ICANS) were the most common adverse events across the trials [8]. These findings underscore the substantial hematopoietic stress imposed by CAR-T therapy in patients with limited marrow reserve.

Patients with multiple myeloma have a significantly increased risk of developing both therapy-induced and nontherapy-induced MDS, with estimates suggesting up to an 11-fold higher risk compared to the general population [9]. In managing RRMM, CAR-T therapy has demonstrated excellent remission rates [5]. While short-term complications such as cytopenias, CRS, and ICANS are well documented, long-term complications remain less understood. One concerning long-term complication is the development of secondary primary malignancies, including MDS [10]. In the CARTITUDE-1 study, after a median follow-up of 28 months, 6.25% of heavily pretreated RRMM patients receiving cilta-cel developed MDS [11]. When acute myeloid leukemia (AML) cases are included, the rate increases to 9.3%. At this time, the data indicates a correlation between CAR-T therapy and secondary malignancies, not a proven causal relationship.

In this context, our patient had previously received lenalidomide, cyclophosphamide, and myeloablative chemotherapy with melphalan before undergoing autologous stem cell transplantation, all of which have been linked to secondary hematologic malignancy including MDS [12]. A retrospective study published in 2022 found that thalidomide and proteasome inhibitors were directly associated with therapy-related myeloid neoplasm [13]. Furthermore, they supported their hypothesis through clustered regularly interspaced short palindromic repeats (CRISPR)-based mutagenesis in murine hematopoietic stem and progenitor cells. Lenalidomide has been shown to selectively promote the expansion of TP53-mutant clones, whereas pomalidomide does not, suggesting a selective advantage for TP53-mutant clones that may be unique to lenalidomide [13]. Fortunately, the patient had a bone marrow biopsy with NGS prior to lymphodepleting chemotherapy, showing no evidence of dysplastic changes or myeloid clones, supporting the possibility that the MDS may not be related to prior therapies. Another proposed mechanism involves the expansion of pre-existing myeloid clones below the limit of detection by NGS following lymphodepleting chemotherapy and CAR-T therapy. These findings highlight the potential importance of performing bone marrow evaluations both pre- and post-CAR-T therapy to assess the bone marrow for the current status of disease along with potential secondary pathology that should be monitored. Additionally, our patient received ide-cel rather than cilta-cel. While preliminary data suggest ide-cel may be associated with a lower risk of MDS, there are yet to be head-to-head trials comparing safety profiles [14,15]. 

A phase 1 clinical trial investigated HBI0101, a novel BCMA-targeting CAR-T therapy for RRMM. Among 40 heavily pretreated multiple myeloma patients, 10% developed MDS after a median follow-up of six months, highlighting a concerning trend in secondary malignancies associated with CAR-T therapy [16]. It is important to note that all four of these patients had MDS-related mutations prior to beginning CAR-T therapy without evidence of morphological MDS. Analysis of their bone marrow aspirate after receiving CAR-T revealed no new MDS-related mutations but instead showed a significantly increased allelic frequency and mutation load of these genetic abnormalities. This suggests that the development of MDS following CAR-T may be due to the expansion of pre-existing myeloid clones rather than the de novo development of MDS-related mutations. In a post hoc study examining a phase 1 trial of 62 heavily pretreated multiple myeloma patients receiving ide-cel, two patients developed MDS. However, upon further analysis, both patients developed MDS strongly associated with their previous alkylating therapy [17]. Overall, based off the current available data, this suggests that MDS following CAR-T therapy may reflect clonal evolution within a previously treated marrow environment, though definitive causality remains difficult to establish.

In evaluating alternative etiologies for this patient's MDS, it is important to recognize that prior myeloma therapies, including induction regimens, maintenance regimens, and myeloablative chemotherapy for autologous transplant, are all recognized contributors to therapy-related hematologic malignancies, including MDS. Although this patient was previously exposed to lenalidomide, current evidence does not support lenalidomide as a strong causative agent in MDS pathogenesis. A 2014 meta-analysis of seven randomized trials in newly diagnosed multiple myeloma demonstrated that lenalidomide is associated with an increased incidence of therapy-related MDS, but this risk is largely confined to patients receiving concurrent oral melphalan [18]. At five years, hematologic second primary malignancy (SPM) occurred in 3.1% of lenalidomide-treated patients compared with 1.4% of controls, with the excess risk driven almost entirely by the lenalidomide/melphalan combination (HR approximately 4.9 versus melphalan alone). In contrast, lenalidomide used without melphalan did not show a significant increase in MDS or AML risk. This data supports that lenalidomide is not the primary cause of MDS; the risk mainly comes from the use with alkylating agents like melphalan. Epidemiology data indicate that the dominant drivers of therapy-related MDS are DNA-damaging cytotoxic agents and ionizing radiation. A 2018 retrospective analysis utilizing the Center for International Blood and Marrow Transplant Research (CIBMTR) and Surveillance, Epidemiology, and End Results (SEER) data demonstrated that therapy-related MDS arises through mutational pathways distinct from de novo disease and is strongly associated with prior exposure to therapy. In the autologous transplant population, 335 post-transplant AML/MDS cases were identified (3.7%), with the highest risk observed in patients who received total body irradiation-based conditioning, underwent multiple prior chemotherapy lines, or received recent transplants. Overall, the incidence of post-transplant AML/MDS was substantially elevated, approximately a 100-fold increase for MDS [19]. 

The mechanism by which CAR-T therapy may contribute to the development of MDS remains unclear. Patients receiving CAR-T therapy often have a history of extensive treatment with alkylating agents and immunomodulatory therapies, both of which are well-established risk factors for MDS [20]. Future studies are essential to elucidate the cellular mechanisms underlying this relationship and to determine which CAR-T therapies offer the safest profiles and if they truly cause secondary malignancy or merely a means for a pre-existing clone to potentiate.

## Conclusions

Our case describes a patient with MDS secondary to CAR-T therapy for RRMM. As CAR-T therapy is becoming more standard of care for the management of RRMM, the long-term complications, such as the development of secondary hematologic malignancy, need to be better understood. We propose multiple biological mechanisms for the development of MDS including the expansion of an undetected pre-existing clone prior to CAR-T and bone marrow injury from prior chemotherapy; therefore, establishing causality will be difficult if not impossible at this time. Further studies will be needed to understand this relationship and investigate if there are other mechanisms not yet identified that establish causality. At present, there are no standardized guidelines for the surveillance of malignancy post-CAR-T including MDS, and the true incidence of MDS post-CAR-T remains unknown at this time. This burden will knowingly fall on the community oncologist to follow their patients closely. While a single case does not establish causality, it lays the foundation for further clinical trials evaluating the safety of CAR-T to allow for more informed clinical decision-making.
